# Inhibition of proteasomal deubiquitinases USP14 and UCHL5 overcomes tyrosine kinase inhibitor resistance in chronic myeloid leukaemia

**DOI:** 10.1002/ctm2.1038

**Published:** 2022-09-09

**Authors:** Liling Jiang, Qingyan He, Xin Chen, Aochu Liu, Wa Ding, Haichuan Zhang, Xinmei Chen, Huan Zhou, Yi Meng, Bingyuan Liu, Guanjie Peng, Chunyan Wang, Jinbao Liu, Xianping Shi

**Affiliations:** ^1^ Guangzhou Municipal and Guangdong Provincial Key Laboratory of Protein Modification and Degradation Affiliated Cancer Hospital of Guangzhou Medical University, State Key Laboratory of Respiratory Disease, School of Basic Medical Sciences, Guangzhou Medical University Guangzhou P.R. China; ^2^ The Sixth Affiliated Hospital of Guangzhou Medical University Qingyuan People's Hospital Qingyuan P.R. China; ^3^ Depatrment of Hematology the First Affiliated Hospital of Guangzhou Medical University Guangzhou Medical University Guangzhou P.R. China

**Keywords:** b‐AP15, BCR‐ABL^T315I^, CML, UCHL5, USP14

## Abstract

**Background:**

Chronic myeloid leukaemia (CML) is a haematological cancer featured by the presence of BCR‐ABL fusion protein with abnormal tyrosine kinase activation. Classical tyrosine kinase inhibitor (TKI)‐based therapies are available to patients with CML. However, acquired resistance to TKI has been a challenging obstacle, especially stubborn T315I mutation is the most common cause. Therefore, it is especially urgent to find more effective targets to overcome TKI resistance induced by BCR‐ABL^T315I^. Proteasomal deubiquitinases (USP14 and UCHL5) have fundamental roles in the ubiquitin‐proteasome system and possess multiple functions during cancer progression.

**Methods:**

The human peripheral blood mononuclear cells were collected to measure the mRNA expression of USP14 and UCHL5, as well as to detect the toxicity effect of b‐AP15. We explored the effect of b‐AP15 on the activity of proteasomal deubiquitinases. We detected the effects of b‐AP15 on BCR‐ABL^WT^ and BCR‐ABL^T315I^ CML cells in vitro and in the subcutaneous tumour model. We knocked down USP14 and/or UCHL5 by shRNA to explore whether these proteasomal deubiquitinases are required for cell proliferation of CML.

**Results:**

In this study, we found that increased expression of the proteasomal deubiquitinase USP14 and UCHL5 in primary cancer cells from CML patients compared to healthy donors. b‐AP15, an inhibitor of USP14 and UCHL5, exhibited potent tumour‐killing activity in BCR‐ABL^WT^ and BCR‐ABL^T315I^ CML cell lines, as well as in CML xenografts and primary CML cells. Mechanically, pharmacological or genetic inhibition of USP14 and UCHL5 induced cell apoptosis and decreased the protein level of BCR‐ABL in CML cells expressing BCR‐ABL^WT^ and BCR‐ABL^T315I^. Moreover, b‐AP15 synergistically enhanced the cytotoxic effect caused by TKI imatinib in BCR‐ABL^WT^ and BCR‐ABL^T315I^ CML cells.

**Conclusion:**

Collectively, our results demonstrate targeting USP14 and UCHL5 as a potential strategy for combating TKI resistance in CML.

## Graphical abstract

1. b‐AP15, an inhibitor of proteasomal deubiquitinases USP14 and UCHL5, induces cell apoptosis and downregulates BCR‐ABL signaling in BCR‐ABL^WT^ and BCR‐ABL^T315I^ CML cell lines and primary CML cells.
2. Silencing of USP14 and UCHL5 suppresses cell proliferation and decreases BCR‐ABL expression in CML cells.
3. USP14 and UCHL5 are potential targets for combating T315I mutant‐triggered TKI resistance in CML.

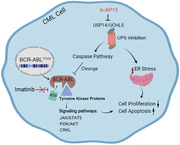

## 1 BACKGROUND

Chronic myeloid leukaemia (CML) is a haematopoietic malignancy, mainly caused by the formation of fusion gene BCR‐ABL, which is generated from t(9;22) chromosome translocation.^1^ Therefore, selective BCR‐ABL tyrosine kinase inhibitor (TKI) is a classic strategy for CML therapy. Nevertheless, the T315I gatekeeper mutant impairs the binding between TKI and the ATP‐pocket of BAR‐ABL, conferring cell resistant to first‐ and second‐generation TKI (e.g. imatinib, nilotinib and dasatinib) in clinics.^2^ In order to override this type of mutation, a third‐generation TKI, ponatinib, has been exploited. However, ponatinib's severe cardiovascular toxicity profile limits its clinical application.^3^ Thus, additional strategies to overcome T315I resistance are desperately needed.

Since tumour cells are characterised by abnormal metabolism, the ubiquitin‐proteasome system (UPS) that mainly functions in intracellular protein turnover is highly active. A paramount example of this problem is the observation that specific inhibitors targeting the UPS represents an effective strategy for cancer therapy.^4^, ^5^ The integrated 26S proteasome is comprised of two 19S regulation portions and a core 20S degradation portion. Before an ubiquitinated protein is degraded by the proteasome, it should be firstly recruited to 19S portion, where its polyubiquitin chain is removed by 19S proteasomal deubiquitinases, including USP14, UCHL5 and POH1.^6^ The first 20S proteasome inhibitor, bortezomib, has been approved by FDA for the treatment of multiple myeloma.^7^, ^8^ However, acquired resistance of bortezomib hampers its therapeutic efficacy. Fortunately, pharmacological inhibition of proteasomal deubiquitinases provides an alternative strategy to interfering with protein degradation by the proteasome.^6^ Therefore, proteasomal deubiquitinases are now considered as promising candidates for antitumour strategy.^9^

b‐AP15 is an inhibitor of proteasomal deubiquitinases, including USP14 and UCHL5.^10^ The antineoplastic effects of b‐AP15 have been shown to be related to activation of acute proteotoxic stress response and production of reactive oxygen species.^11^, ^12^, ^13^, ^14^ Accordingly, b‐AP15 shows a significant antineoplastic effect in both solid and nonsolid tumours, including oesophageal squamous cell carcinoma, neuroblastoma and multiple myeloma.^15^, ^16^, ^17^, ^18^ However, the effects of proteasomal deubiquitinase inhibitor on CML have not yet been reported.

In this study, we revealed the potential of targeting proteasomal deubiquitinase as a therapeutic strategy in TKI‐resistant CML. We demonstrated that targeting USP14 and UCHL5 by b‐AP15 showed obvious antitumour efficiency in BCR‐ABL^WT^ and BCR‐ABL^T315I^ CML cell lines and xenografts, as well as in primary CML cells. Furthermore, we found that b‐AP15 synergised imatinib to induce cell death in TKI‐resistant CML cells. These findings have important implications for improving therapies aimed at overcoming TKI resistance in CML.

## 2 MATERIALS AND METHODS

### 2.1 Cell lines and PBMCs

KBM5 cells expressed BCR‐ABL^WT^ was isolated from a CML patient in blast crisis.^19^ KBM5‐T315I cells were derived from KBM5 by long‐term exposing to imatinib and selecting the survival clones harbouring the BCR‐ABL^T315I^.^20^ BaF3 stably expressing BCR‐ABL^WT^ or BCR‐ABL^T315I^ were kindly provided by B.Z Carter (University of Texas M.D. Anderson Cancer Center, USA). K562 cells were purchased from ATCC. KBM5 and KBM5‐T315I were cultured in IMDM medium with 10% FBS. BaF3, BaF3‐T315I, and K562 cells were cultured in RPMI 1640 medium with 10% FBS.

PBMCs were collected from the Hematology Department, the First Affiliated Hospital of Guangzhou Medical University, which was approved by the medical research ethics committee review (approve number: 2022–77), with the permission of the patients. PBMCs were isolated and cultured in RPMI 1640 medium with 15% FBS.

### 2.2 Chemicals and reagents

The small molecules b‐AP15, ZVAD, imatinib and bortezomib were purchased from Selleck Chemicals and dissolved in DMSO and stored at –20°C. The final dose of DMSO limited to 0.3% in all experiments. The antibodies as follows were obtained from Cell Signaling Technology (Danvers, MA): PARP (#9532S), p27 (#3688S), USP14 (#11931S), Caspase‐3 (#9662S), Caspase‐8 (#9746S), Caspase‐9 (#9508S), XIAP (#2042S), Bax (#5023), Bcl‐2 (15071S), Survivin (#2808S), AIF (#4642S), Cytochrome C (#4272S), PERK (#5683S), ATF4 (#11815S), CHOP (#2895), BCR‐ABL (#2862S), p‐BCR‐ABL (#2861S), STAT5 (#9363S), p‐AKT (#2965), AKT (#4685), CRKL (#3182S) and p‐CRKL (#3181S). The antibodies as follows were obtained from Proteintech (Rosemont, USA): Mcl‐1 (#16225‐1‐Ap) and BCL‐xl (#66020‐I‐Ig). The antibodies as follows are purchased from Abcam (Cambridge, MA): Cleaved Caspase 9 (#AB3629) and Cleaved Caspase 3 (#AB2302). The antibody of ubiquitin was purchased from Santa Cruz Biotechnology. p‐STAT5 (#50095), anti‐mouse (12‐349) and anti‐rabbit (AP132P) antibodies were obtained from Merk Millipore (Darmstadt, Germany). All the above antibodies were used at a dilution of 1:1000.

### 2.3 Western blots

Western blots were performed as previously described.^20^ In brief, cells were collected and washed twice with PBS. Whole‐cell lysate is generated using RIPA buffer (Beyotime Biotechnology, Shanghai, China) supplemented with PMSF and Protease Inhibitor Cocktail (Roche, Indianapolis, IN). The bicinchoninic acid (BCA) assay was used to determine the total protein concentration.

### 2.4 qPCR

Total RNA was isolated with QIAsymphony RNA Kit (QIAGEN, Germantown, MD), 1 µg RNA used for cDNA synthesis by RNA reverse PCR Kit (TaKaRa, Dalian, Shangdong). Diluted the cDNA as 1:10, then prepare reaction system following the protocol of SYBR Premix Ex TaqII Kit (TaKaRa), then measured in Bio‐Red CFX‐96 Touch Real‐Time PCR system. GAPDH was used as endogenous control and mRNA expression of genes was analysed by the Comparative Ct method. The primers are as follows:

USP14 forward, 5′‐CAGCTGTTTGCGTTGACTGG‐3′
USP14 reverse, 5′‐GAAGAGCATCTGCTGACCCC‐3′
UCHL5 forward, 5′‐GGCTCTGTGGTTCAGGACTC‐3′
UCHL5 reverse, 5′‐TGGACATCCTGGTGGGTACA‐3′
POH1 forward, 5′‐GAGGCAAGACAAGGGTCCATC‐3′
POH1 reverse, 5′‐TTCTGGCAGGTACAACTTCCC‐3′

### 2.5 Proteasomal activity assay

In brief, cells were lysed in assay buffer (25 mM Tris‐HCl, pH 7.4), and then incubated with b‐AP15 or imatinib on ice for 30 min. To evaluate the proteasome's chymotrypsin‐like (CT‐like), caspase‐like (C‐like), and trypsin‐like activity (T‐like), cell lysis was incubated with Suc‐LLVY‐AMC, or Z‐LLE‐AMC or Boc‐LRR‐AMC for 2 h in the dark at 37°C.

### 2.6 HA‐Ub‐VS assay

HA‐Ub‐VS is a probe targeting the deubiquitinase active site and can be used to test the binding capacity of compounds to deubiquitinase. Cells were harvested after incubated 3 h with b‐AP15, lysed in a lysis buffer (25 mM Tris‐HCl, 20 mM NaCl, 5 mM MgCl_2_, 200 µM ATP, pH7.4) for 30 min. The equal amounts of protein from each sample were incubated with HA‐Ub‐VS (1 µM) in 37°C for 30 min. Finally, the HA‐Ub‐VS labelled proteins were measured by Western blots.

### 2.7 Cell viability

A total of 2 × 10^4^ cells/well were seeded in 96 well plates with a volume of 100 µl and were stimulated with b‐AP15 for 48 h and incubated with 20 µl MTS (Promega, Madison, WI) at 37°C for 2 h. Then, the absorbance was measured by a microplate reader at wavelength 490 nm.

### 2.8 Cell apoptosis

Cell apoptosis was investigated using Annexin V/PI double staining kit (Sungene Biotech, Tianjin, China). The cells were harvested after the treatment of b‐AP15 for 24 h, and washed twice with ice‐cold PBS, followed by incubation on ice for 5 min with 5 µl Annexin V. Then, the cells were incubated with PI for 10 min, followed with a flow cytometer and Cytexpert software analysis.

### 2.9 Mitochondrial membrane permeability

The mitochondrial membrane potential was determined by rhodamine 123 (Merck, Darmstadt, Germany) staining. Briefly, cells were stimulated with b‐AP15 for 24 h, and collected for rhodamine 123 staining. Then, cells were resuspended in 500 µl cell culture medium, and incubated with 2.5 µg/ml rhodamine 123 for 30 min at 37°C in the dark. Finally, the mitochondrial membrane potential of cells was measured by flow cytometer and analysed by Cytexpert software.

### 2.10 Xenograft model

Five‐week‐old nude Balb/c mice were raised and housed in the animal facility of Guangzhou Medical University. Inoculated subcutaneously on the mice with 1 × 10^7^ cells of CML cells, every cell type includes 12 mice. The mice were randomly separated into a vehicle and b‐AP15 after inoculated, and then intraperitoneal injection with vehicle (20% Cremophor EL, 20% PEG400, and 40% Saline) or 5 mg/kg/day b‐AP15 every day. The mice body weight and tumour size were recorded, and the tumour volume was calculated as previously described.^20^ The mice were sacrificed when the maximum tumour reached 1500 mm^3^, then the tumour tissue that was isolated from the mice body and weighted. The tumour tissue was lysed for western blot analysis or fixed for immunohistochemical staining. The experiments were subjected to the approval of the Institutional Animal Care and Use Committee of Guangzhou Medical University and following the ethical rules of animal experiments (GY2019‐109).

### 2.11 Immunohistochemical staining

4% paraformaldehyde‐fixed xenografts were used to prepare the paraffin section according to a standard method. Paraffin sections were immunostained for K48‐linkage ubiquitin, Ki67 and BCR‐ABL. The freshly prepared DAB solution (Beyotime, Shanghai, China) was used to detect the reaction products. Then, haematoxylin was used to counterstain the section.

### 2.12 Analysis of drug synergistic effect

Increasing concentrations of b‐AP15 were co‐treatment with IC_(30)_ or IC_(50)_ of imatinib (IM), the cell viability were measured by MTS assay. CompuSyn software was used to calculate the combination index (CI) which is a representative of the combined effects of two drugs.^21^, ^22^ The CI value less than, equal to, and greater than 1 indicates a synergistic, additive, and antagonistic effect, respectively.

### 2.13 Statistical analysis

Each experiment was repeated at least three times, and the graphs were all shown mean ± SD. *p* < .05 was considered statistically significant. Statistics were performed using Graphpad Prism 7.0 software. One‐way ANOVA followed by Tukey's test was adopted for multiple groups comparison.

## 3 RESULTS

### 3.1 b‐AP15 suppresses the deubiquitinase activity of USP14 and UCHL5 and induces cytotoxic effect in BCR‐ABL^WT^ and BCR‐ABL^T315I^ CML cells

**FIGURE 1:**
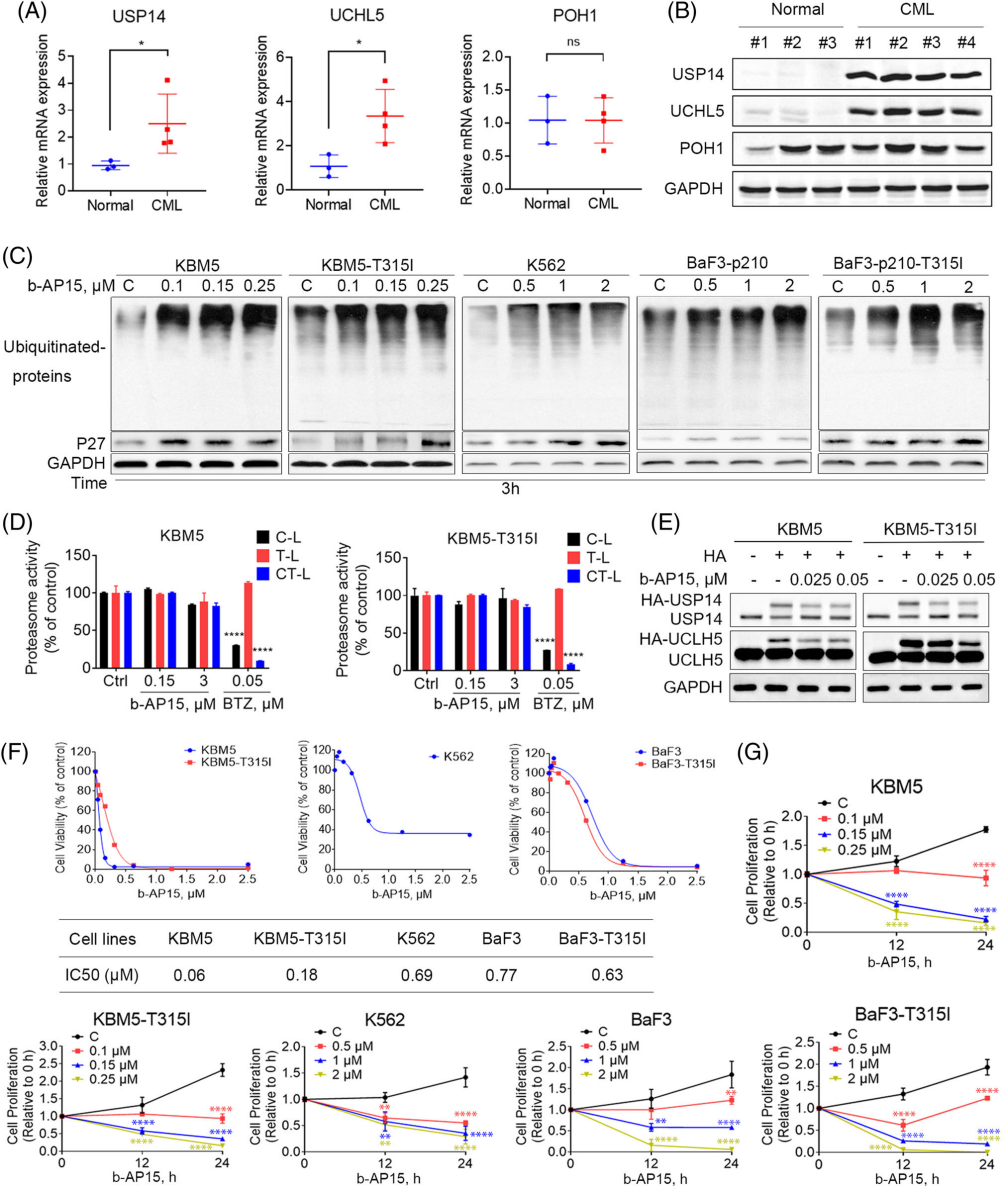
b‐AP15 inhibits the cell viability of BCR‐ABL^WT^ and BCR‐ABL^T315I^ CML cells through targeting USP14 and UCHL5. (A) The mRNA expression of the indicated proteasomal deubiquitinases in PBMCs from CML patients and healthy donors. Mean±SD (*n* = 3), *p < .05, versus normal group. ns: no significance. (B) The protein level of the indicated proteasomal deubiquitinases was determined by Western blots. (C) b‐AP15 induced the accumulation of ubiquitinated proteins and p27 in CML cells. (D) The effects of b‐AP15 on the hydrolase activity of 20S proteasome in CML cells. Mean ± SD (*n* = 3), ****p < .0001, versus control group. (E) b‐AP15 inhibited the deubiquitinase activity of USP14 and UCHL5 in CML cells. CML cells were treated with b‐AP15 or DMSO for 3 h. The cell lysates were labelled with HA‐Ub‐VS, followed by immunoblotting with USP14 and UCHL5 antibodies. (F) MTS assay was performed to measure the cell viability of CML cells incubated with b‐AP14 for 48 h. (G) Cell counting after trypan blue staining of CML cells treated with b‐AP15. ****p < .0001, versus control group

To establish the clinical relevance of tumour proteasomal deubiquitinases expression in CML patients, the expression of proteasomal deubiquitinases (USP14, UCHL5 and POH1) was examined in peripheral blood mononuclear cells (PBMCs) from CML patients as well as that from healthy donors. We found that the mRNA and protein level of USP14 and UCHL5 in PBMCs from CML patients was significantly higher than that from healthy donors (Figure 1A and B). However, the expression level of POH1 in CML patients was similar to that in normal human (Figure 1A and B). In addition, we have tested the USP14 and UCHL5 protein level in BCR‐ABL^WT^ (KBM5 and BaF3) versus BCR‐ABL^T315I^ (KBM5‐T315I and BaF3‐T315I) cells. But there were no significant differences between BCR‐ABL^WT^ and BCR‐ABL^T315I^ CML cells (Figure S1A). b‐AP15, an inhibitor of USP14 and UCHL5, has been reported to impair the function of UPS.^10^ Thus, we analysed the UPS inhibitory effects of b‐AP15 in BCR‐ABL^WT^ (KBM5, K562, and BaF3) and BCR‐ABL^T315I^ (KBM5‐T315I and BaF3‐T315I) CML cells. As expected, b‐AP15 increased the levels of ubiquitinated proteins and p27, a protein routinely degraded by the proteasome (Figure 1C). In addition, we investigated the effects of b‐AP15 on the level of DVL2 protein, which is a well‐established substrate of proteasomal deubiquitinase.^23^ As expected, the results showed that b‐AP15 enhanced the level of DVL2 protein, indicating the suppression of proteasome function (Figure S1B). However, unlike the 20S proteasome inhibitor bortezomib, b‐AP15 did not affect the C‐, T‐, and CT‐like enzymatic activities of 20S proteasome (Figure 1D). Furthermore, we performed an in vitro deubiquitinase activity assay with HA‐Ub‐VS, which binds to deubiquitinase active sites. We found that b‐AP15 decreased HA‐Ub‐VS labelled USP14 and UCHL5 (Figure 1E), indicating that b‐AP15 inhibited the activities of proteasomal deubiquitinases in CML cells.

Next, the cytotoxic effect of b‐AP15 was tested in a panel of TKI‐sensitive and ‐resistant CML cells. We observed that b‐AP15 suppressed the cell viability of BCR‐ABL^WT^ and BCR‐ABL^T315I^ CML cells (Figure 1F). Moreover, trypan blue dye exclusion assay showed that b‐AP15 suppressed the cell proliferation of BCR‐ABL^WT^ and BCR‐ABL^T315I^ CML cells (Figure 1G). Collectively, these findings illustrate that inhibition of proteasomal deubiquitinases by b‐AP15 kills TKI‐sensitive and ‐resistant CML cells.

### 3.2 b‐AP15 triggers caspase‐dependent apoptosis in BCR‐ABL^WT^ and BCR‐ABL^T315I^ CML cells

**FIGURE 2:**
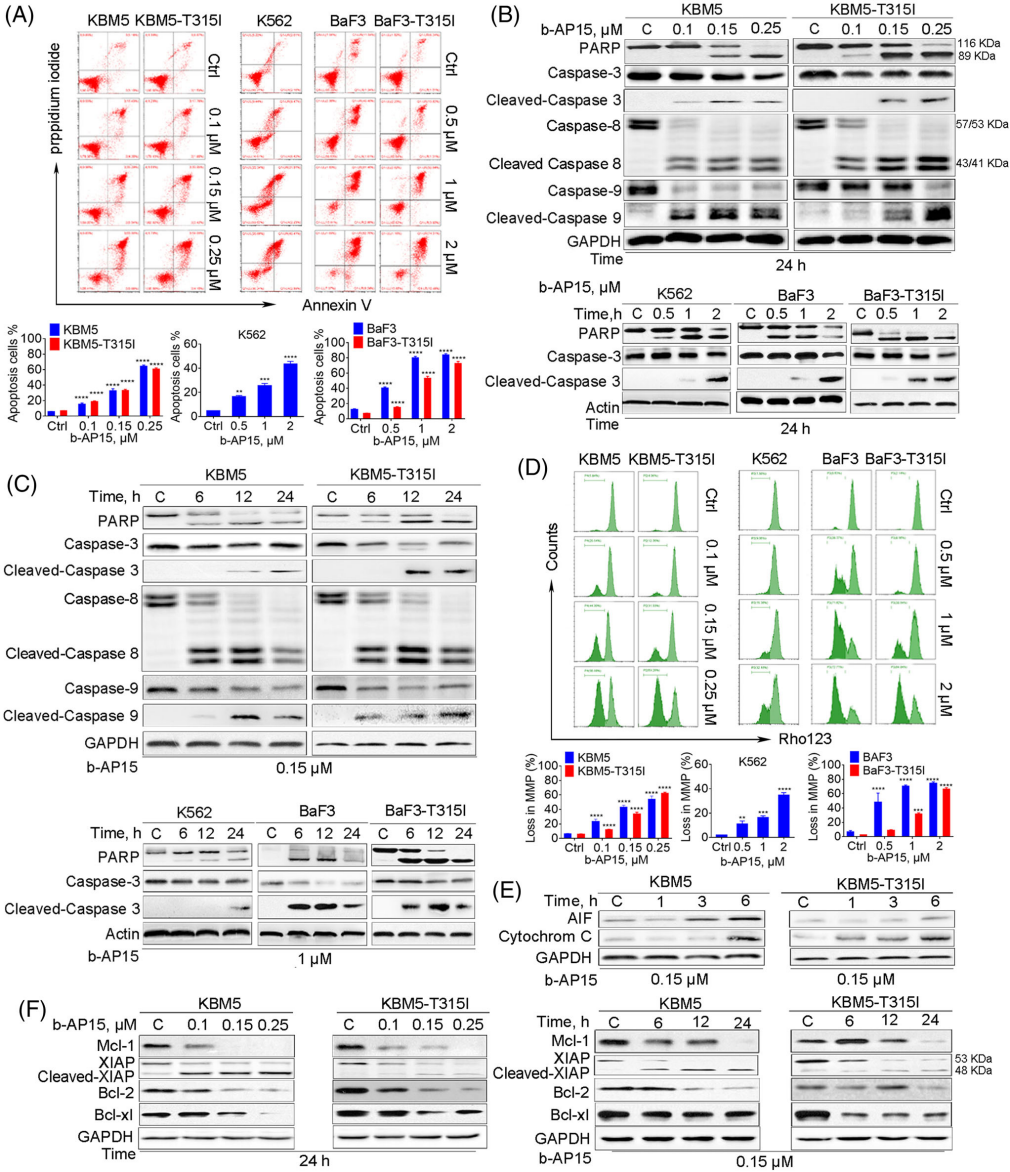
b‐AP15 induces cell apoptosis in BCR‐ABL^WT^ and BCR‐ABL^T315I^ CML cells. (A) CML cells were incubated with b‐AP15 for 24 h, and then Annexin V/PI double staining was performed to detect cell apoptosis. Mean ± SD (*n* = 3), **p < .01, ***p < .001, ****p < .0001, versus control group. (B), (C) CML cells were treated with b‐AP15, and then the indicated proteins were detected by Western blots. (D) CML cells were treated with b‐AP14 for 24 h, and then mitochondrial membrane potential was detected by rhodamine 123 assay and flow cytometry. Mean ± SD (*n* = 3), **p < .01, ***p < .001, ****p < .0001, versus control group. (E) The protein level of AIF and Cytochrome C in the cytoplasm segment of KBM5 and KBM5‐T315I cells was detected by Western blots. (F) KBM5 and KBM5‐T315I cells were stimulated with b‐AP15, and then the indicated proteins were detected by Western blots

To explore the mechanisms of b‐AP15‐induced cytotoxic effect, we performed the annexin V/propidium iodide assay to evaluate cell death. The results showed that b‐AP15 induces cell apoptosis in BCR‐ABL^WT^ and BCR‐ABL^T315I^ CML cells (Figure 2A). Apoptosis is mainly mediated by caspase‐mediated cleavage cascade.^24^ To verify whether b‐AP15 activates the classical caspase signalling pathway, we examined the cleaved counterparts of caspase‐3, ‐8, ‐9 in BCR‐ABL^WT^ and BCR‐ABL^T315I^ CML cells. We found that b‐AP15 caused activation of caspases, as well as cleavage of PARP (Figure 2B and C). b‐AP15 also caused a decrease in mitochondrial membrane potential (Figure 2D), which has been linked to the induction of apoptosis. Consistently, mitochondrial AIF and cytochrome C proteins were released into the cytosol in b‐AP15‐treated CML cells (Figure 2E), suggesting b‐AP15 induced the opening of mitochondrial pore transition. Furthermore, b‐AP15 induced downregulation of anti‐apoptosis proteins, including Bcl‐2, Mcl‐1, and Bcl‐xl in BCR‐ABL^WT^ and BCR‐ABL^T315I^ CML cells (Figure 2F). Likewise, b‐AP15 induced the cleavage of XIAP, which may reflect the activation of caspases. These results strongly support that b‐AP15 kills TKI‐sensitive and ‐resistant CML cells via caspase‐dependent apoptosis.

### 3.3 b‐AP15 activates ER stress and suppresses BCR‐ABL signallings in CML cells

**FIGURE 3:**
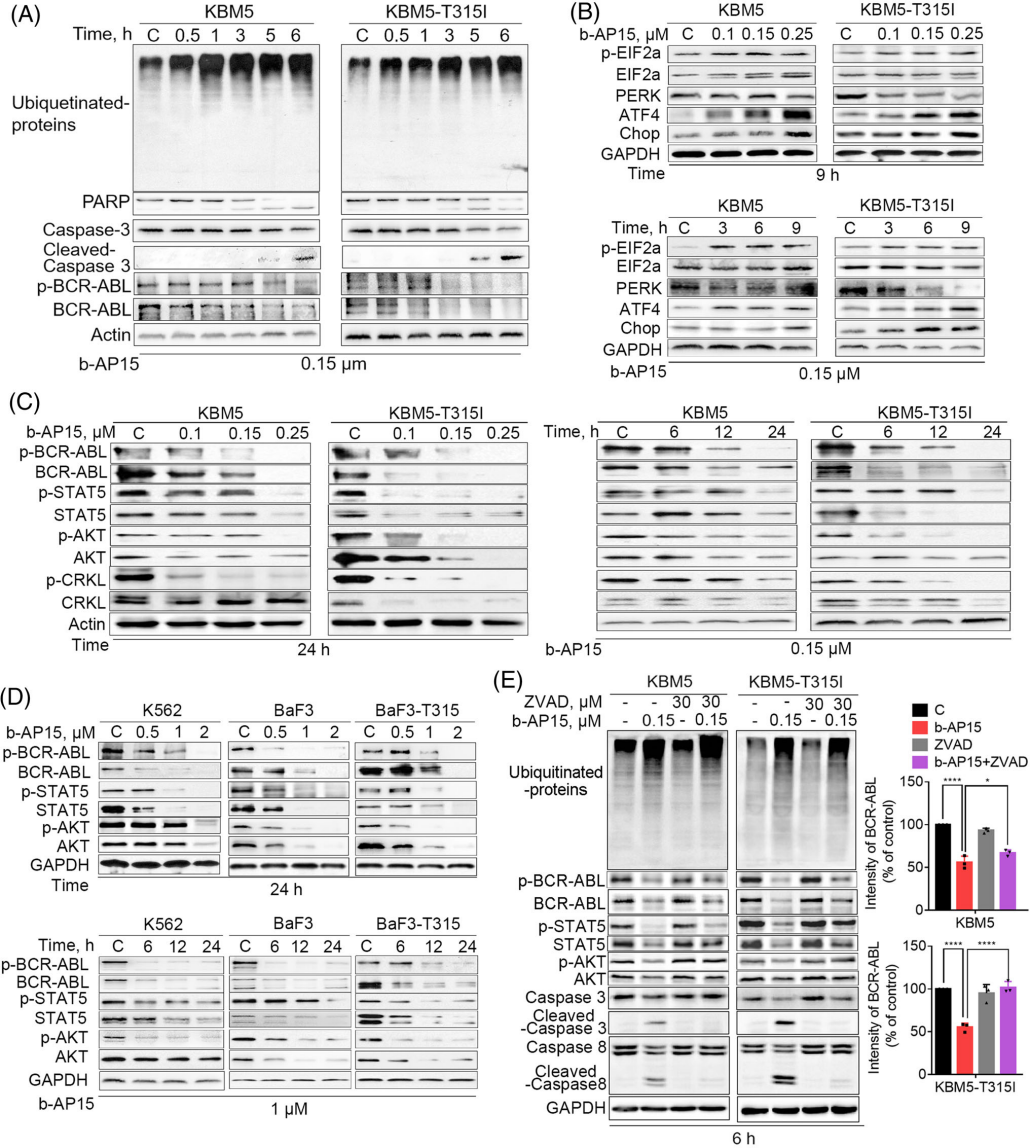
b‐AP15 activates ER stress and downregulates BCR‐ABL signallings. (A)–(D) CML cells (KBM5, KBM5‐T315I, K562, BaF3 and BaF3‐T315) were treated with b‐AP15, and then the indicated proteins were detected by Western blots. (E) KBM5 and KBM5‐T315I cells were incubated with 0.15 µM b‐AP15 with or without 30 µM ZVAD, and then the indicated proteins were measured by Western blots. The histogram indicates the abundance of BCR‐ABL. *p < .05, ****p < .0001

To explore the relationship between proteasome inhibition and apoptosis, we investigated the markers of proteasome inhibition and cell apoptosis at sequential time points. Importantly, the accumulation of ubiquitinated proteins occurred significantly earlier than PARP cleavage in CML cells treated with b‐AP15 (Figures 3A), suggesting that apoptosis is triggered after UPS inhibition by b‐AP15. It has shown that dysfunction of UPS results in intracellular ER stress through the accumulation of misfolded proteins.^25^ Indeed, b‐AP15 increased the expression of p‐EIF2ɑ, ATF4 and CHOP, while it inhibited the expression of PERK (Figure 3B), which are hallmarks of ER stress. Moreover, we have detected ER stress at earlier time points and found that ER stress was activated at 0.5 h, a time point without visible apoptosis (PARP cleavage) (Figure S1C). Thus, ER stress is indeed a sensitive indicator of proteasome inhibition. Furthermore, we found that TUDCA (ER stress inhibitor) partially reduced b‐AP15‐induced apoptosis in KBM5 and KBM5‐T315I cells (Figure S1D). These results demonstrate that b‐AP15 triggers ER stress to activates cell apoptosis by proteasome inhibition.

Given the key role of BCR‐ABL in the malignant progression of CML, we tested the effect of b‐AP15 on BCR‐ABL‐related signallings. As shown in Figure 3C and D, BCR‐ABL and its phosphorylation was decreased in b‐AP15‐treated BCR‐ABL^WT^ and BCR‐ABL^T315I^ cells. Furthermore, the cascade signalling pathways of BCR‐ABL, including STAT5, AKT and CRKL, were also suppressed by b‐AP15 (Figure 3C and D). Next, we investigated how did b‐AP15 induce downregulation of BCR‐ABL. Our and other group have demonstrated that BCR‐ABL can be cleaved by caspases.^20^, ^26^. Thus, we examined whether activation of the caspase pathway is responsible for b‐AP15‐induced downregulation of BCR‐ABL. We observed that co‐treatment with z‐VAD‐fmk (a caspase inhibitor) inhibited the downregulation of BCR‐ABL and the cascade signalling pathways of BCR‐ABL in CML cells following the treatment with b‐AP15 (Figure 3E). However, there is no change in ubiquitinated proteins (Figure 3E), indicating that UPS inhibition may be an upstream event to the downregulation of BCR‐ABL signallings. These results indicate that b‐AP15‐induced activation of caspases is partially essential for BCR‐ABL downregulation in TKI‐sensitive and ‐resistant CML cells.

### 3.4 Pharmacological or genetic targeting of USP14 and UCHL5 induces cytotoxic effect in CML cells

**FIGURE 4:**
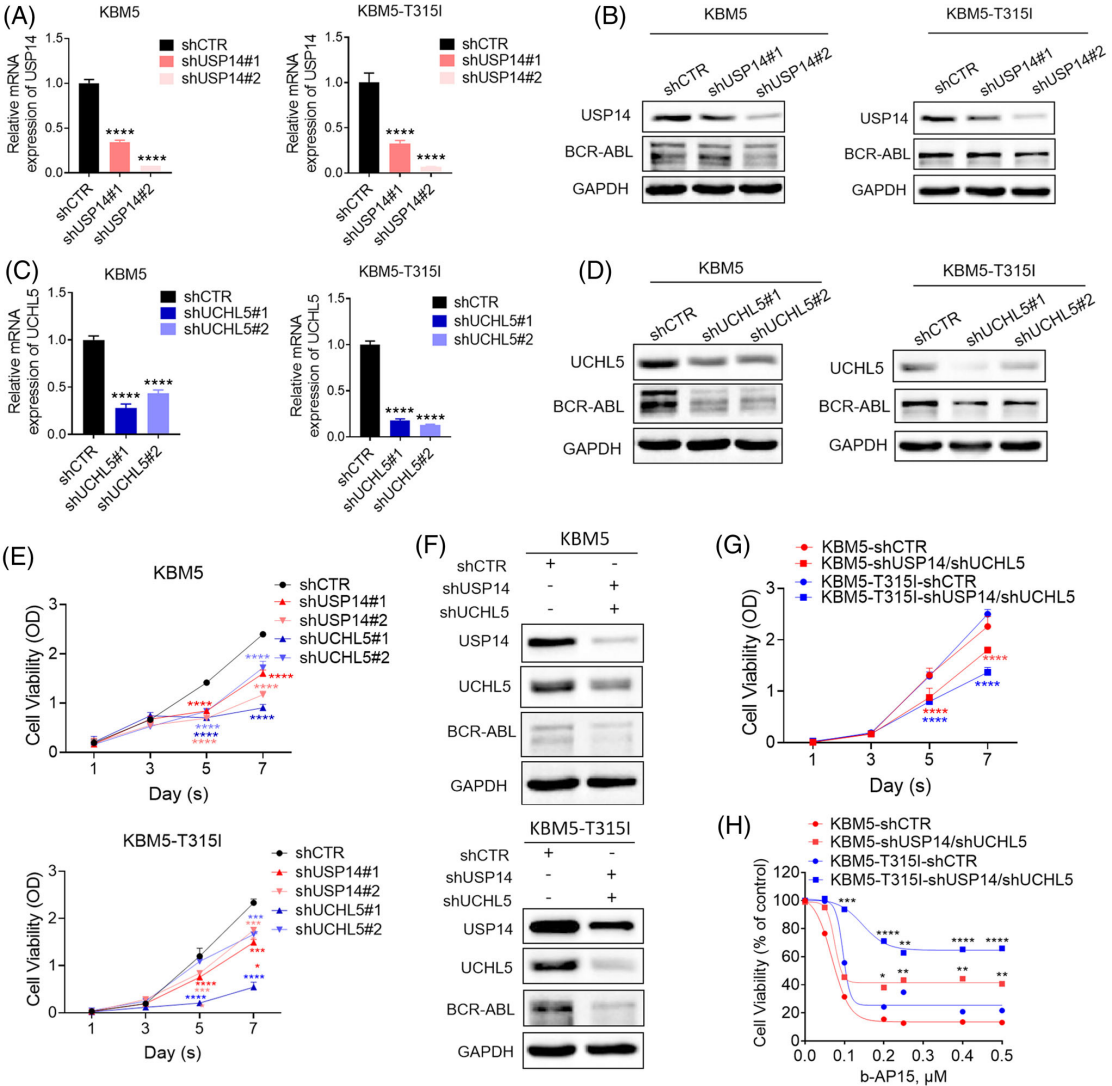
Knockdown of USP14 or/and UCHL5 attenuates b‐AP15‐induced cytotoxic effect in CML cells. (A–E) KBM5 or KBM5‐T315I cells were stably transfected with either control siRNA (Random), human USP14 shRNA (shUSP14 #1 and shUSP14 #2), or human UCHL5 shRNA (shUCHL5 #1 and shUCHL5 #2). (A), (C). The mRNA expression of USP14 or UCHL5 genes in the indicated CML cells. Mean ± SD (*n* = 3), ****p < .0001, versus control group. (B), (D). The indicated proteins were detected by Western blot analysis. E. The cell viability of the indicated cells was measured by MTS assay. Mean ± SD (*n* = 3). ****p < .0001, versus control group. (F)–(H) KBM5 or KBM5‐T315I cells were stably transfected with either control shRNA (shCTR) or human USP14 and UCHL5 shRNA (shUSP14/shUCHL5). (F) The indicated proteins were detected by Western blots. (G), (H) The indicated cells were incubated with increasing concentrations of b‐AP15 for 48 h, and then the cell viability was measured by MTS assay. Mean ± SD (*n* = 3). **p < .01, ***p < .001, ****p < .0001, versus control group

To test whether USP14 and UCHL5 maintain cell proliferation of CML, we knocked down USP14 and/or UCHL5 in KBM5 and KBM5‐T315I cells and observed that knockdown of either USP14 or USPL5 alone decreased BCR‐ABL expression and CML cell viability (Figure 4A–E). Moreover, the combined knockdown of USP14 and UCHL5 induced a robust decrease in BCR‐ABL protein level and cell viability of CML cells (Figure 4F and G). Importantly, the combined knockdown of USP14 and UCHL5 attenuated b‐AP15‐induced cytotoxic effect in CML cells (Figure 4H), further confirming that USP14 and UCHL5 indeed act as direct target of b‐AP15. Consistently, Hinokitiol copper complex (HKCu), another reported USP14 and UCHL5 inhibitor,^27^ also decreased cell viability in CML cells (Figure S1E). Moreover, we overexpressed USP14 and/or UCHL5 in KBM5 and KBM5‐T315I cells and found that overexpression of USP14 and/or UCHL5 inhibited the anti‐proliferative effects of b‐AP15 in KBM5 and KBM5‐T315I cells (Figure S1F and G). In conclusion, these findings demonstrate that the inhibition of USP14 and UCHL5 may kill TKI‐sensitive and ‐resistant CML cells through downregulating BCR‐ABL levels.

### 3.5 b‐AP15 suppresses the growth of CML xenografts

**FIGURE 5:**
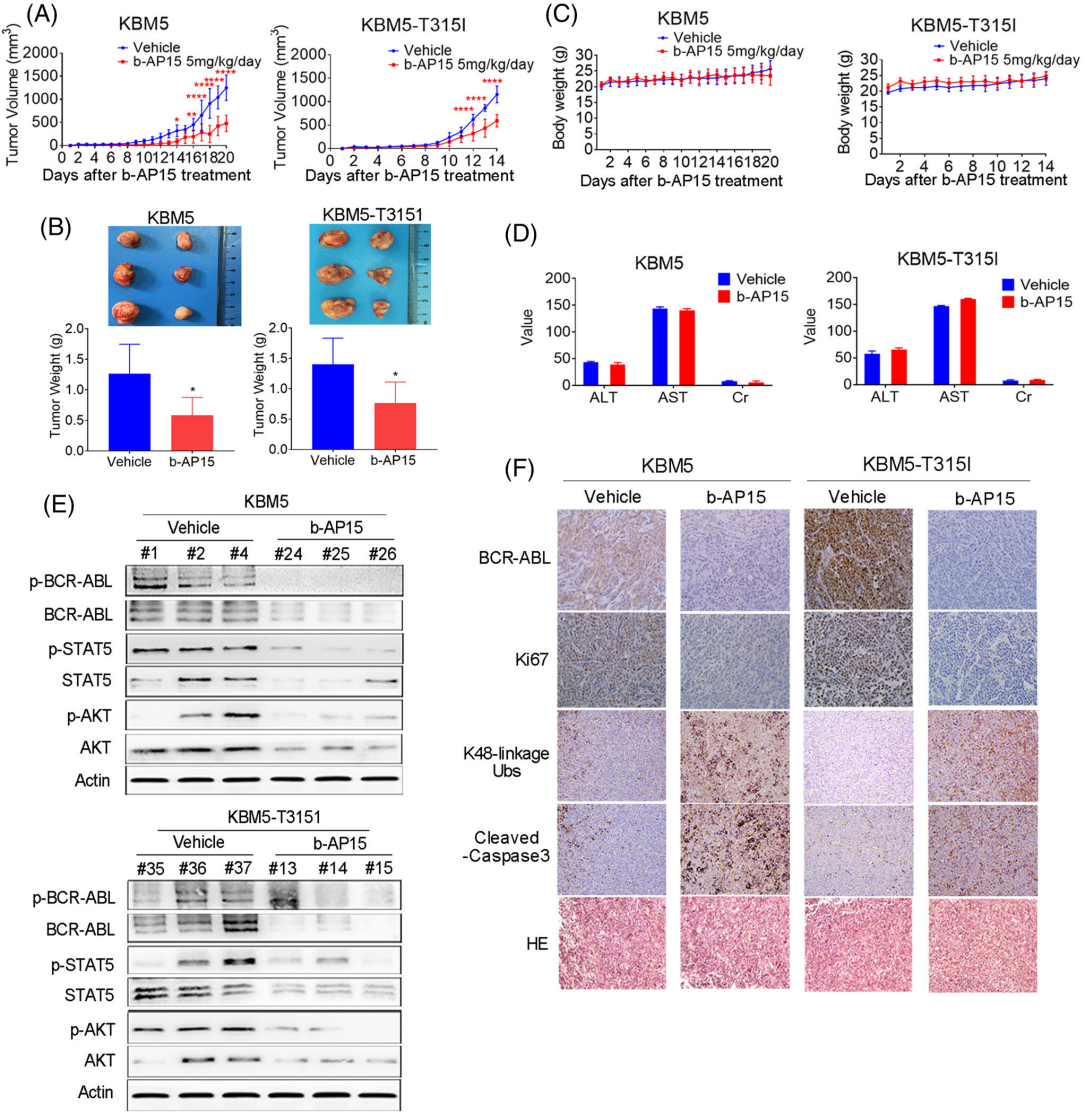
b‐AP15 suppresses the growth of CML xenografts. (A) CML (KBM5 and KBM5‐T315I) xenograft bearing nude mice were treated with vehicle or b‐AP15 (5mg/kg/day) since tumour size reached 50 mm^3^, and then the tumour sizes were recorded every day. (B) At the terminal of the experiments, the mice were sacrificed, and then the tumours were excised and weighed. (C) The body weight of mice was shown. (D) The serum ALT, AST and Cr levels were measured by a blood test. (E) The indicated proteins in tumour tissue were detected by Western blots. (F) Immunohistochemical (IHC) analysis of the indicated proteins in tumour tissue. Mean ± SD (*n* = 6), *p < .05, ****p < .0001, versus vehicle group

To explore the antitumour effect of b‐AP15 in vivo, KBM5 and KBM5‐T315I cells were injected into the subcutis of nude mice. Every cohort was randomly separated into vehicle group and b‐AP15 treatment group. Continuous b‐AP15 treatment caused prolonged inhibition of tumour growth of CML xenografts (Figure 5A). Meanwhile, compared with the vehicle group, both tumour size and tumour weight were decreased in the b‐AP15 group (Figure 5B). However, no significant difference was found in the body weight between two groups (Figure 5C). Likewise, the value of hepatorenal toxicity indicators (e.g. ALT, AST and Cr) were indistinguishable between two groups (Figure 5D). Therefore, b‐AP15 appears to be safe and effective at the tested doses. Moreover, expression of BCR‐ABL, STAT5, AKT and their phosphorylation counterparts were decreased by b‐AP15 treatment (Figure 5E), indicating the tyrosine kinase cascade of BCR‐ABL was suppressed by b‐AP15. Also, the immunohistochemical staining of tumour sections showed an increase of K48‐linkage ubiquitinated proteins and a decrease of BCR‐ABL and Ki67 in the b‐AP15 treatment group compared with the vehicle group (Figure 5F and Figure S1H). Moreover, the increased cleaved‐caspase 3 also observed in IHC assay (Figure 5F). The results corroborate that b‐AP15 exhibits potent in vivo efficacy with a favourable safety profile for the treatment of CML.

### 3.6 b‐AP15 induces cell apoptosis in PBMCs from CML patients

**FIGURE 6:**
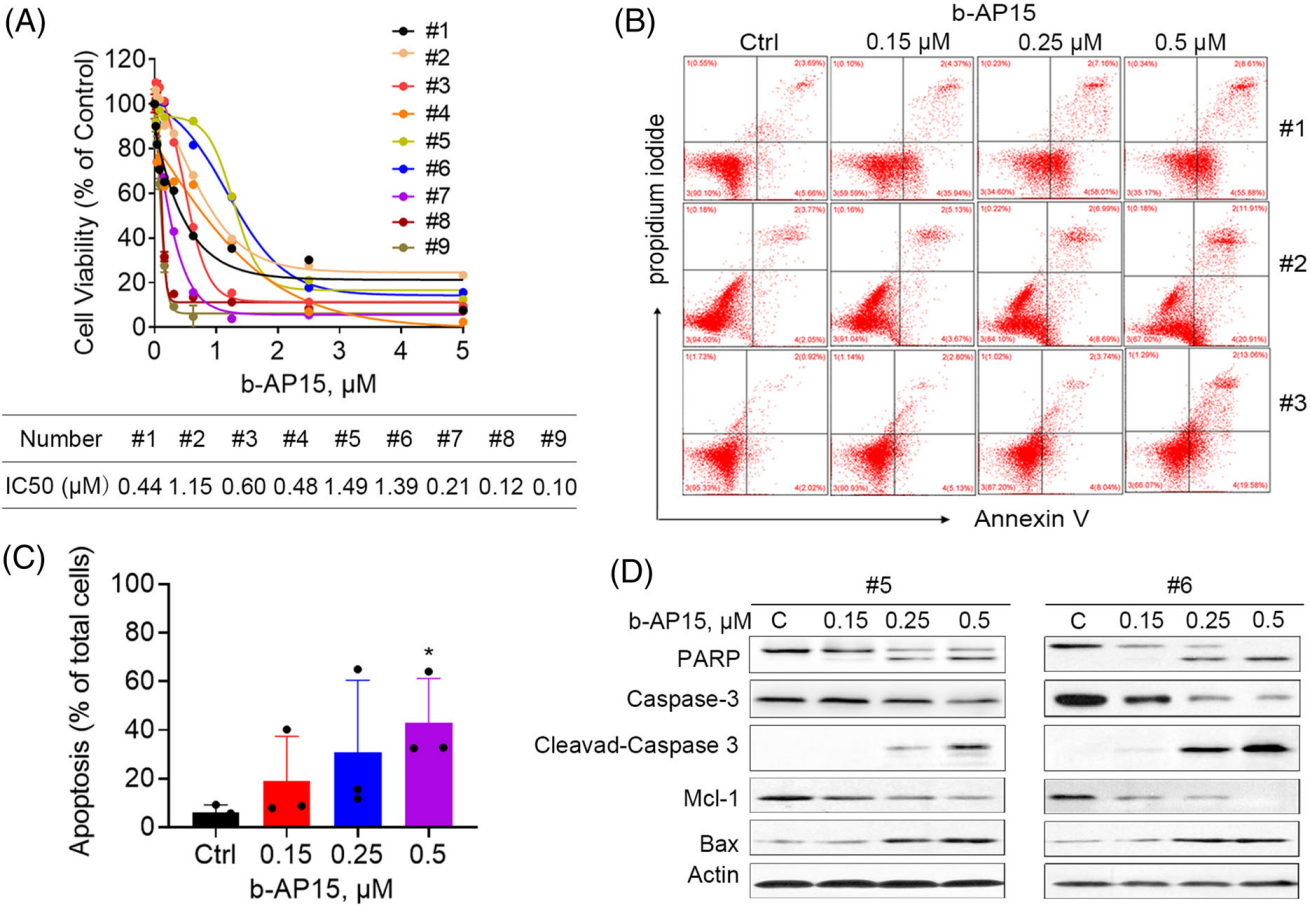
b‐AP15 induces cell apoptosis in PBMCs from CML patients. (A) PBMCs from CML patients were treated with increasing doses of b‐AP15 for 48 h, and then cell viability was measured by MTS assay. (B), (C) PBMCs from CML patients were treated with b‐AP15 for 24 h, then apoptosis was detected by Annexin V/PI double staining and cytometry analysis. The graphs were the statistics of the Annexin V^+^ cells. Mean ± SD (*n* = 4). *p < .05, versus control group. (D) PBMCs from CML patients were treated with b‐AP15 for 24 h, and then the indicated proteins were measured by Western blots

To further verify the activity of b‐AP15 in CML cells and provide research evidence for its clinical application, we explored the biological function of b‐AP15 on PBMCs from BCR‐ABL^WT^ (#1‐#7) and BCR‐ABL^MUT^ (#8 and #9) CML patients. The clinical and biological characteristics of CML samples collected in this study were shown in Table S1. In this regard, b‐AP15 suppressed the cell viability of primary CML cells, with IC_50_ ranged from 0.1 to 1.49 µM (Figure 6A). Similar to the results of cultured cell lines, b‐AP15 triggered cell apoptosis in these primary CML cells (Figure 6B and C). Moreover, b‐AP15 induced cleavage of PARP and caspase 3, as well as downregulation of Mcl‐1 and upregulation of Bax (Figure 6D), further supporting the anti‐cancer activities of b‐AP15 in CML. It worth noting that PBMCs from healthy donors was relatively tolerant to b‐AP15, with IC_50_ of 5.8–10.4 µM, as we reported previously.^28^ These results indicate that b‐AP15 may serve as an efficient and safe anticancer agent for CML patients.

### 3.7 b‐AP15 and imatinib synergistically induce cell apoptosis in BCR‐ABL^WT^ and BCR‐ABL^T315I^ cells

**FIGURE 7:**
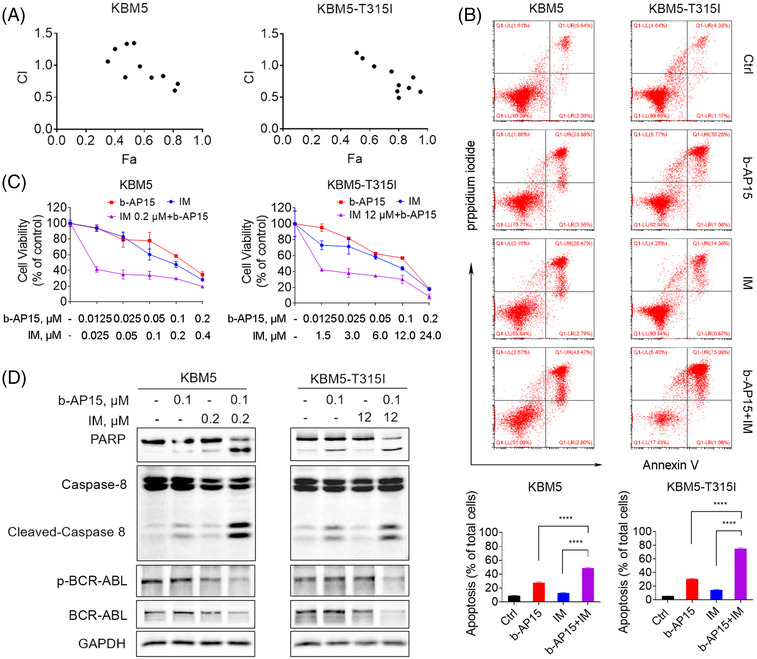
b‐AP15 and imatinib synergistically induce cell apoptosis in CML cells. (A) The CI values of b‐AP15 combined with imatinib in KBM5 and KBM5‐T315I cells were shown. CI = 1 means additive effect, CI < 1 means synergism, and CI > 1 means antagonism. Fraction affected (Fa) is the proportion of cells affected by combination treatment. (B) KBM5 and KBM5‐T315I cells was treated with b‐AP15 and/or imatinib, and then cell viability was measured by MTS assay. (C), (D) KBM5 cell were treated with b‐AP15 (0.1 µM) and/or imatinib (0.2 µM), KBM5‐T315I cells were treated with b‐AP15 (0.1 µM) and/or imatinib (12 µM), then cell apoptosis was detected after 24 h by Annexin V/PI double staining, and the indicated proteins were measured by Western blots. The graphs were the statistics of the Annexin V^+^ cells. Mean ± SD (*n* = 3). ****p < .0001, versus control group

Since b‐AP15 showed a significant reduction effect on BCR‐ABL, we speculated a synergistic effect between tyrosine kinase inhibitor imatinib and proteasomal deubiquitinase inhibition. To verify this hypothesis, we performed MTS assay to examine the cell viability and calculated the combination index based on their concentration and biological activity. The results showed that the combination index values of imatinib and b‐AP15 at most doses were lower than 1 in BCR‐ABL^WT^ and BCR‐ABL^T315I^ CML cells, indicating a synergism between imatinib and b‐AP15 (Figure 7A). Imatinib in combination with b‐AP15 dramatically reduced cell viability when compared with cells treated with either agent alone (Figure 7B). Similarly, knockdown of USP14 and UCHL5 enhanced imatinib reduced cell viability in CML cells (Figure S1I). Combined treatment with imatinib and b‐AP15 also resulted in a synergistic apoptosis‐ inducing effect in KBM5 and KBM5‐T315I cells (Figure 7C). Furthermore, cleaved caspase‐8 and PARP proteins were increased in the combined group, while BCR‐ABL protein and its phosphorylation were decreased (Figure 7D). These findings confirm the synergistic inhibition of imatinib and b‐AP15 on TKI‐sensitive and ‐resistant CML cells.

## 4 DISCUSSION

Proteasome tightly regulates the degradation of most intracellular proteins, which is a crucial mechanism to maintain cellular homeostasis. There are three proteasomal deubiquitinases USP14, UCHL5, and POH1, which function as a critical step regulates protein degradation through removing the poly‐ubiquitin chain from substrates.^6^ Recent studies have reported that proteasomal deubiquitinases are highly expressed in various cancers, suggesting a potential antineoplastic function of their inhibitors.^29^, ^30^, ^31^ However, the function of proteasomal deubiquitinases in CML has rarely been reported.

The patients with BCR‐ABL‐positive CML can benefit from target therapy with TKI, however the toxicity and side effects, the drug off‐target risks, the resistance, and the distinction of disease molecular profile are the limiting factors for TKIs.^32^ It is pretty important to find the etiological mechanism of TKI resistance. Here, we compared the expression of proteasomal deubiquitinases between healthy donors and CML patients. Both USP14 and UCHL5 were expressed higher in CML patients, suggesting that USP14 and UCHL5 could be a novel target for CML treatment. This was partially consistent with the report that USP14 was highly expressed in CML compared with normal marrow.^33^ Interestingly, several deubiquitinase, such as USP47, is transcriptionally regulated by BCR‐ABL.^33^ However, whether BCR‐ABL transcriptionally controls USP14 or UCHL5 in CML is still unknown. In addition, we demonstrated that knockdown of USP14 and UCHL5 increased imatinib sensitivity and cell apoptosis in BCR‐ABL^WT^ and BCR‐ABL^T315I^ CML cells. These results open up alternative avenues for overcoming imatinib resistance, such as using USP14 and UCHL5 inhibitors as single agents or combined with imatinib in the treatment of T315I mutant CML patients.

b‐AP15 contains an α,β‐unsaturated carbonyl, which reversibly reacts with cysteine proteases USP14 and UCHL5, but not decreases the activities of the 20S proteasome.^34^ It has shown that b‐AP15 rapidly distributes into cells and elicits rapid apoptosis.^34^ We observed that b‐AP15 showed similar effects through targeting USP14 and UCHL5. Moreover, our results also demonstrated that b‐AP15 suppressed cell growth through downregulating the expression of BCR‐ABL in CML cells and CML xenografts. Importantly, b‐AP15 was toxic to PBMCs from CML patients and synergistically induced cell apoptosis with imatinib on BCR‐ABL^WT^ and BCR‐ABL^T315I^ CML cells. Noteworthy, VLX1570, an analogue of b‐AP15, was advanced into Phase I clinical trial, although this project was discontinued due to pulmonary toxicity.^35^ It has been postulated that the adverse events appear to be related to the formulation.^35^ Nevertheless, efforts directed at identifying proteasome deubiquitinase inhibitors carries important clinical potential.

It has been documented that the accumulation of excessive ubiquitinated proteins activates ER stress and ultimately elicits cell death.^36^, ^37^ Recent studies have reported that b‐AP15 and its analogue triggered cell apoptosis via activation of ER stress in leukaemia and solid tumour cells.^38^, ^39^, ^40^ In agreement with these findings, our work revealed that b‐AP15 treatment induced ER stress and cell apoptosis in BCR‐ABL^WT^ and BCR‐ABL^T315I^ CML cells, which may be related to the accumulation of unfolded protein response by inhibiting USP14 and UCHL5. In addition, BCR‐ABL can prevent apoptotic death in CML cells partly by inducing Bcl‐2 and MCl‐1 expression.^41^, ^42^ Our work revealed that b‐AP15 decreased the expression of anti‐apoptosis proteins including Bcl‐2 and Mcl‐1, which may contribute to b‐AP15‐induced cell apoptosis in CML.

It is worth noting that BCR‐ABL was slightly decreased by b‐AP15 stimulation at an earlier time before the presence of cell apoptosis, and it was obviously decreased

at a later time point accompanied by a strong induction of cell death. This relationship indicates that downregulation of BCR‐ABL may be related to the occurrence of apoptosis. Our data demonstrated that BCR‐ABL downregulation partially relied on the activation of caspases, which directly mediated the cleavage of BCR‐ABL and the proteins of key downstream signalling pathway regulated by BCR‐ABL, such as STAT5, p‐STAT5, AKT, and p‐AKT in the same condition. However, other caspase‐independent mechanism may also be involved, which have not yet been elucidated. Recent studies have confirmed that BCR‐ABL can be degraded via the UPS, and several deubiquitinases have been reported to participate in this process. For instance, USP7 and USP25 cleave K48‐linkage poly‐ubiquitination conjugate from BCR‐ABL, which accelerates BCR‐ABL degradation through UPS.^43^, ^44^ In addition, inhibition of USP9X triggers K63‐linkage poly‐ubiquitination of BCR‐ABL, resulting in its accumulation in aggresomes.^45^ It is still unknown whether USP14 or UCHL5 regulates the deubiquitination and subsequent degradation of BCR‐ABL protein. Therefore, further interrogation of the mechanism involved in BCR‐ABL degradation is required before firm conclusions can be drawn.

In conclusion, this study demonstrates that b‐AP15 induces cell apoptosis and downregulates BCR‐ABL in BCR‐ABL^WT^ and BCR‐ABL^T315I^ CML cells (Figure 8). These findings provide a novel understanding of USP14 and UCHL5 as an important therapeutic target for TKI‐resistant CML treatment.

## CONFLICT OF INTEREST

The authors declare that they have no competing interests.

## Supporting information

Supplementary information

## References

[1] Ren R . Mechanisms of BCR‐ABL in the pathogenesis of chronic myelogenous leukaemia. Nat Rev Cancer 2005;5(3):172–183. 15719031 10.1038/nrc1567

[2] Braun TP , Eide CA , Druker BJ . Response and resistance to BCR‐ABL1‐targeted therapies. Cancer Cell 2020;37(4):530–542. 32289275 10.1016/j.ccell.2020.03.006 PMC7722523

[3] Meenakshi Sundaram DN , Jiang X , Brandwein JM , Valencia‐Serna J , Remant KC , Uludag H . Current outlook on drug resistance in chronic myeloid leukemia (CML) and potential therapeutic options. Drug Discov Today 2019;24(7):1355–1369. 31102734 10.1016/j.drudis.2019.05.007

[4] Morgan JJ , Crawford LJ . The ubiquitin proteasome system in genome stability and cancer. Cancers (Basel) 2021;13(9). 10.3390/cancers13092235 PMC8125356 34066546

[5] Hoeller D , Dikic I . Targeting the ubiquitin system in cancer therapy. Nature 2009;458(7237):438–444. 19325623 10.1038/nature07960

[6] D'Arcy P , Linder S . Proteasome deubiquitinases as novel targets for cancer therapy. Int J Biochem Cell Biol 2012;44(11):1729–1738. 22819849 10.1016/j.biocel.2012.07.011

[7] Richardson PG , Hideshima T , Anderson KC . Bortezomib (PS‐341): a novel, first‐in‐class proteasome inhibitor for the treatment of multiple myeloma and other cancers. Cancer Control 2003;10(5):361–369. 14581890 10.1177/107327480301000502

[8] LaPlante G , Zhang W . Targeting the ubiquitin‐proteasome system for cancer therapeutics by small‐molecule inhibitors. Cancers (Basel) 2021;13(12). 10.3390/cancers13123079 PMC8235664 34203106

[9] Mofers A , Pellegrini P , Linder S , D'Arcy P . Proteasome‐associated deubiquitinases and cancer. Cancer Metastasis Rev 2017;36(4):635–653. 29134486 10.1007/s10555-017-9697-6 PMC5721125

[10] D'Arcy P , Brnjic S , Olofsson MH , et al. Inhibition of proteasome deubiquitinating activity as a new cancer therapy. Nat Med 2011;17(12):1636–1640. 22057347 10.1038/nm.2536

[11] Brnjic S , Mazurkiewicz M , Fryknas M , et al. Induction of tumor cell apoptosis by a proteasome deubiquitinase inhibitor is associated with oxidative stress. Antioxid Redox Signal 2014;21(17):2271–2285. 24011031 10.1089/ars.2013.5322 PMC4241954

[12] Hillert EK , Brnjic S , Zhang X , et al. Proteasome inhibitor b‐AP15 induces enhanced proteotoxicity by inhibiting cytoprotective aggresome formation. Cancer Lett 2019;448:70–83. 30768956 10.1016/j.canlet.2019.02.003

[13] Ming SL , Zhang S , Wang Q , et al. Inhibition of USP14 influences alphaherpesvirus proliferation by degrading viral VP16 protein via ER stress‐triggered selective autophagy. Autophagy 2021:1–21. 10.1080/15548627.2021.2002101 PMC9450976 34822318

[14] Xia X , Liao Y , Guo Z , et al. Targeting proteasome‐associated deubiquitinases as a novel strategy for the treatment of estrogen receptor‐positive breast cancer. Oncogenesis 2018;7(9):75. 30250021 10.1038/s41389-018-0086-y PMC6155249

[15] Sha B , Chen X , Wu H , et al. Deubiquitylatinase inhibitor b‐AP15 induces c‐Myc‐Noxa‐mediated apoptosis in esophageal squamous cell carcinoma. Apoptosis 2019;24(9‐10):826–836. 31342239 10.1007/s10495-019-01561-9

[16] Tian Z , D'Arcy P , Wang X , et al. A novel small molecule inhibitor of deubiquitylating enzyme USP14 and UCHL5 induces apoptosis in multiple myeloma and overcomes bortezomib resistance. Blood 2014;123(5):706–716. 24319254 10.1182/blood-2013-05-500033 PMC3907756

[17] Kropp KN , Maurer S , Rothfelder K , et al. The novel deubiquitinase inhibitor b‐AP15 induces direct and NK cell‐mediated antitumor effects in human mantle cell lymphoma. Cancer Immunol Immunother 2018;67(6):935–947. 29556699 10.1007/s00262-018-2151-y PMC11028140

[18] Yu Y , Zhao Y , Fan Y , et al. Inhibition of ubiquitin‐specific protease 14 suppresses cell proliferation and synergizes with chemotherapeutic agents in neuroblastoma. Mol Cancer Ther 2019;18(6):1045–1056. 30962318 10.1158/1535-7163.MCT-18-0146 PMC6565366

[19] Beran M , Pisa P , O'Brien S , et al. Biological properties and growth in SCID mice of a new myelogenous leukemia cell line (KBM‐5) derived from chronic myelogenous leukemia cells in the blastic phase. Cancer Res 1993;53(15):3603–3610. 8339266

[20] Shi X , Chen X , Li X , et al. Gambogic acid induces apoptosis in imatinib‐resistant chronic myeloid leukemia cells via inducing proteasome inhibition and caspase‐dependent Bcr‐Abl downregulation. Clin Cancer Res 2014;20(1):151–163. 24334603 10.1158/1078-0432.CCR-13-1063 PMC3938960

[21] Gencer EB , Ural AU , Avcu F , Baran Y . A novel mechanism of dasatinib‐induced apoptosis in chronic myeloid leukemia; ceramide synthase and ceramide clearance genes. Ann Hematol 2011;90(11):1265–1275. 21455605 10.1007/s00277-011-1212-5

[22] Yenigul M , Akcok I , Gencer Akcok EB . Ethacrynic acid and cinnamic acid combination exhibits selective anticancer effects on K562 chronic myeloid leukemia cells. Mol Biol Rep 2022;49(8):7521–7530. 10.1007/s11033-022-07560-5 35585382

[23] Jung H , Kim BG , Han WH , et al. Deubiquitination of Dishevelled by Usp14 is required for Wnt signaling. Oncogenesis 2013;2: e64. 23958854 10.1038/oncsis.2013.28 PMC3759127

[24] Carneiro BA , El‐Deiry WS . Targeting apoptosis in cancer therapy. Nat Rev Clin Oncol 2020;17(7):395–417. 32203277 10.1038/s41571-020-0341-y PMC8211386

[25] Qu J , Zou T , Lin Z . The roles of the ubiquitin‐proteasome system in the endoplasmic reticulum stress pathway. Int J Mol Sci 2021;22(4). 10.3390/ijms22041526 PMC7913544 33546413

[26] Lan X , Zhao C , Chen X , et al. Nickel pyrithione induces apoptosis in chronic myeloid leukemia cells resistant to imatinib via both Bcr/Abl‐dependent and Bcr/Abl‐independent mechanisms. J Hematol Oncol 2016;9(1):129. 27884201 10.1186/s13045-016-0359-x PMC5123219

[27] Chen X , Zhang X , Chen J , et al. Hinokitiol copper complex inhibits proteasomal deubiquitination and induces paraptosis‐like cell death in human cancer cells. Eur J Pharmacol 2017;815:147–155. 28887042 10.1016/j.ejphar.2017.09.003

[28] Jiang L , Sun Y , Wang J , et al. Proteasomal cysteine deubiquitinase inhibitor b‐AP15 suppresses migration and induces apoptosis in diffuse large B cell lymphoma. J Exp Clin Cancer Res 2019;38(1):453. 31694672 10.1186/s13046-019-1446-y PMC6836452

[29] Lv C , Wang S , Lin L , Wang C , et al. USP14 maintains HIF1‐alpha stabilization via its deubiquitination activity in hepatocellular carcinoma. Cell Death Dis 2021;12(9):803. 34420039 10.1038/s41419-021-04089-6 PMC8380251

[30] Zhang J , Xu H , Yang X , et al. Deubiquitinase UCHL5 is elevated and associated with a poor clinical outcome in lung adenocarcinoma (LUAD). J Cancer 2020;11(22):6675–6685. 33046988 10.7150/jca.46146 PMC7545677

[31] Seo D , Jung SM , Park JS , et al. The deubiquitinating enzyme PSMD14 facilitates tumor growth and chemoresistance through stabilizing the ALK2 receptor in the initiation of BMP6 signaling pathway. EBioMedicine 2019;49:55–71. 31685442 10.1016/j.ebiom.2019.10.039 PMC7113187

[32] Ciftciler R , Haznedaroglu IC . Tailored tyrosine kinase inhibitor (TKI) treatment of chronic myeloid leukemia (CML) based on current evidence. Eur Rev Med Pharmacol Sci 2021;25(24):7787–7798. 34982440 10.26355/eurrev_202112_27625

[33] Lei H , Xu HZ , Shan HZ , et al. Targeting USP47 overcomes tyrosine kinase inhibitor resistance and eradicates leukemia stem/progenitor cells in chronic myelogenous leukemia. Nat Commun 2021;12(1):51. 33397955 10.1038/s41467-020-20259-0 PMC7782553

[34] Wang X , Stafford W , Mazurkiewicz M , et al. The 19S Deubiquitinase inhibitor b‐AP15 is enriched in cells and elicits rapid commitment to cell death. Mol Pharmacol 2014; 85(6): 932–945. 24714215 10.1124/mol.113.091322

[35] Rowinsky EK , Paner A , Berdeja JG , et al. Phase 1 study of the protein deubiquitinase inhibitor VLX1570 in patients with relapsed and/or refractory multiple myeloma. Invest New Drugs 2020;38(5):1448–1453. 32125598 10.1007/s10637-020-00915-4 PMC7497669

[36] Corazzari M , Gagliardi M , Fimia GM , Piacentini M . Endoplasmic reticulum stress, unfolded protein response, and cancer cell fate. Front Oncol 2017;7:78. 28491820 10.3389/fonc.2017.00078 PMC5405076

[37] Alasiri G , Fan LY , Zona S , et al. ER stress and cancer: The FOXO forkhead transcription factor link. Mol Cell Endocrinol 2018;462(Pt B):67–81. 28572047 10.1016/j.mce.2017.05.027

[38] Ding Y , Chen X , Wang B , Yu B , Ge J . Deubiquitinase inhibitor b‐AP15 activates endoplasmic reticulum (ER) stress and inhibits Wnt/Notch1 signaling pathway leading to the reduction of cell survival in hepatocellular carcinoma cells. Eur J Pharmacol 2018;825:10–18. 29454609 10.1016/j.ejphar.2018.02.020

[39] Kurozumi N , Tsujioka T , Ouchida M , et al. VLX1570 induces apoptosis through the generation of ROS and induction of ER stress on leukemia cell lines. Cancer Sci 2021; 112(8):3302–3313. 34032336 10.1111/cas.14982 PMC8353915

[40] Pellegrini P , Selvaraju K , Faustini E , et al. Induction of ER stress in acute lymphoblastic leukemia cells by the deubiquitinase inhibitor VLX1570. Int J Mol Sci 2020;21(13). 10.3390/ijms21134757 PMC7369842 32635430

[41] Li QF , Huang WR , Duan HF , Wang H , Wu CT , Wang LS . Sphingosine kinase‐1 mediates BCR/ABL‐induced upregulation of Mcl‐1 in chronic myeloid leukemia cells. Oncogene 2007;26(57):7904–7908. 17599053 10.1038/sj.onc.1210587

[42] Sanchez‐Garcia I , Grutz G . Tumorigenic activity of the BCR‐ABL oncogenes is mediated by BCL2. Proc Natl Acad Sci U S A 1995;92(12):5287–5291. 7777499 10.1073/pnas.92.12.5287 PMC41679

[43] Shibata N , Ohoka N , Tsuji G , et al. Deubiquitylase USP25 prevents degradation of BCR‐ABL protein and ensures proliferation of Ph‐positive leukemia cells. Oncogene 2020;39(19):3867–3878. 32203161 10.1038/s41388-020-1253-0

[44] Jiang S , Wang X , He Y , et al. Suppression of USP7 induces BCR‐ABL degradation and chronic myelogenous leukemia cell apoptosis. Cell Death Dis 2021;12(5):456. 33963175 10.1038/s41419-021-03732-6 PMC8105359

[45] Sun H , Kapuria V , Peterson LF , et al. Bcr‐Abl ubiquitination and Usp9x inhibition block kinase signaling and promote CML cell apoptosis. Blood 2011;117(11):3151–3162. 21248063 10.1182/blood-2010-03-276477

