# Supplementary material for: Inhibition of proteasomal deubiquitinases USP14 and UCHL5 overcomes tyrosine kinase inhibitor resistance in chronic myeloid leukaemia
Source: Clin Transl Med. 2022 Sep 9;12(9):e1038. doi: 10.1002/ctm2.1038 (PMC9460481; doi:10.1002/ctm2.1038)
Supplement: Supplementary file 1 — Supplementary information [file CTM2-12-e1038-s001.docx]

**Supplementary Table 1. Characteristics of patients with chronic myeloid leukemia**

| Patient No | Age (yr)/ Sex | Imatinib Resistance | BCR-ABL Mutantion | WBC |
| --- | --- | --- | --- | --- |
| #1 | 56/Male | No | No | 110×10^9^ |
| #2 | 43/Male | No | No | 335.8×10^9^ |
| #3 | 37/Male | No | No | 315.5×10^9^ |
| #4 | 41/Female | No | No | 142.7×10^9^ |
| #5 | 26/Male | No | No | 45×10^9^ |
| #6 | 56/Female | No | No | 123×10^9^ |
| #7 | 47/Male | No | No | 98×10^9^ |
| #8 | 62/Male | Yes | T315I;M244V;L387M | 36.69×10^9^ |
| #9 | 52/Male | Yes | T315I | 1.82×10^9^ |


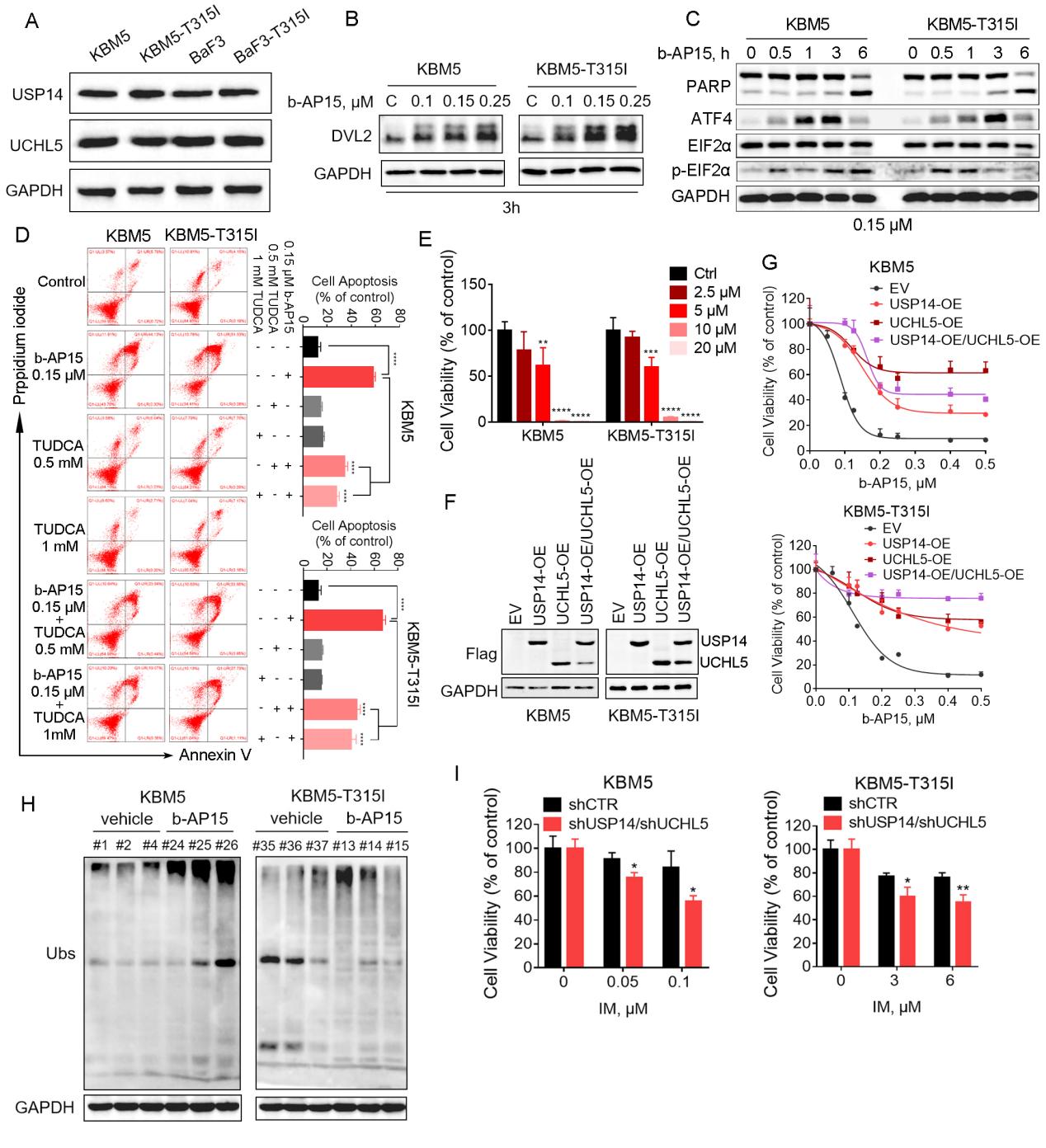


**Supplementary Figure 1. (A) Expression of USP15 and UCHL5 in CML cells.** The indicated proteins of CML cells (KBM5, KBM5T315I, BAF3, and BAF3T315I ) were detected by Western blot analysis. **(B) The effects of b-AP15 on the level of DVL2 protein, which is a well-established substrate of proteasomal deubiquitinase.** The protein level of Dvl2 was detected after b-AP15 treatment in CML cells. **(C)** **b-AP15 induces ER stress in CML cells.** KBM5 and KBM5-T315I cells were treated with b-AP15, and then the indicated proteins were detected by Western blot analysis. **(D) TUDCA reduced b-AP15-triggered apoptosis in KBM5 and KBM5-T315I cells.** KBM5 and KBM5-T315I cells were incubated with 0.15 μM b-AP15 with or without TUDCA, and then the indicated proteins were measured by Western blots. **(E)** **USP14 and UCHL5 inhibitor Hinokitiol copper complex induces cytotoxic effect in CML cells.** KBM5 and KBM5-T315I cells were treated with escalating doses of Hinokitiol copper complex (HKCu) for 48 hours, and then cell viability was measured by MTS assay. **P < 0.01, ***P < 0.001, ****P < 0.0001, versus control group. **(F-J) KBM5 or KBM5-T315I cells were stably transfected with either empty vector of pCDH (EV), pCDH-homo-USP14-flag (USP14-OE), or pCDH-homo-UCHL5-flag (UCHL5-OE).** F. Indicated proteins were detected by Western blot analysis. J. The indicated cells were incubated with increasing concentrations of b-AP15 for 48 h, and then the cell viability was measured by MTS assay. **(H) b-AP15 induce proteasome inhibition in CML xenografts.** Western blot analysis of the indicated proteins in tumor tissue of figure 5. **(I) USP14 and UCHL5 knockdown CML cells is sensitive to imatinib.** KBM5 and KBM5-T315I control or USP14/UCHL5 knockdown cells was treated with imatinib (IM), and then cell viability was measured by MTS assay. *P < 0.05, **P < 0.01, versus control group.
